# Comparative genomics analysis of *bHLH* genes in cucurbits identifies a novel gene regulating cucurbitacin biosynthesis

**DOI:** 10.1093/hr/uhac038

**Published:** 2022-02-19

**Authors:** Yuanchao Xu, Huimin Zhang, Yang Zhong, Naiyu Jiang, Xiaoyun Zhong, Qiqi Zhang, Sen Chai, Hongbo Li, Zhonghua Zhang

**Affiliations:** 1 Key Laboratory of Biology and Genetic Improvement of Horticultural Crops of the Ministry of Agriculture, Sino-Dutch Joint Laboratory of Horticultural Genomics, Institute of Vegetables and Flowers, Chinese Academy of Agricultural Sciences, Beijing 100081, China; 2 Engineering Laboratory of Genetic Improvement of Horticultural Crops of Shandong Province, College of Horticulture, Qingdao Agricultural University, Qingdao 266109, China; 3 Shenzhen Branch, Guangdong Laboratory for Lingnan Modern Agriculture, Shenzhen Key Laboratory of Agricultural Synthetic Biology, Genome Analysis Laboratory of the Ministry of Agriculture and Rural Affairs, Agricultural Genomics Institute at Shenzhen, Chinese Academy of Agricultural Sciences, Shenzhen 518120, China

## Abstract

The basic helix–loop–helix (bHLH) family of transcription factors (TFs) participate in a variety of biological regulatory processes in plants, and have undergone significant expansion during land plant evolution by gene duplications. In cucurbit crops, several *bHLH* genes have been found to be responsible for agronomic traits such as bitterness. However, the characterization of *bHLH* genes across the genomes of cucurbit species has not been reported, and how they have evolved and diverged remains largely unanswered. Here we identified 1,160 *bHLH* genes in seven cucurbit crops and performed a comprehensive comparative genomics analysis. We determined orthologous and paralogous *bHLH* genes across cucurbit crops by syntenic analysis between or within species. Orthology and phylogenetic analysis of the tandem-duplicated *bHLH* genes in the *Bt* cluster, which regulate the biosynthesis of cucurbitacins, suggest that this cluster is derived from three ancestral genes after the cucurbit-common tetraploidization event. Interestingly, we identified a new conserved cluster paralogous to the *Bt* cluster that includes two tandem *bHLH* genes, and the evolutionary history and expression profiles of these two genes in the new cluster suggest the involvement of one gene (*Brp*) in the regulation of cucurbitacin biosynthesis in roots. Further biochemical and transgenic assays in melon hairy roots supported the function of *Brp*. This study provides useful information for further investigating the functions of bHLH TFs and novel insights into the regulation of cucurbitacin biosynthesis in cucurbit crops and other plants.

## Introduction

Basic helix–loop–helix (bHLH) transcription factors (TFs) are widespread in eukaryotes and constitute one of the largest TF families in plants [1]. Many studies suggest that the variety of cucurbit-common *bHLH* genes in plants were derived from one or a few predecessors through a significant number of gene duplications [2–4]. Segmental and tandem duplications or whole-genome duplications (WGDs) should contribute to the expansion of copy numbers in the bHLH family [5, 6]. In addition to the core-eudicot common hexaploidization (ECH) event, cucurbit crops also underwent an ancient cucurbit-common tetraploidization (CCT) event at ~90 million years ago (MYA) [7], and *Cucurbita* species have a recent WGD [8]. Which *bHLH* genes remained after these multiple duplication events and how they have evolved have not been investigated in cucurbit crops.

bHLH TFs can act as transcriptional activators or repressors and play essential roles in plant developmental and physiological processes, such as the regulation of flag leaf angle [9] and shoot branching [10], and responses to light [11], phytohormones [12], and low temperature [13]. Notably, many bHLH TFs regulate the biosynthesis of specialized secondary metabolites in plants [14–19], including cucurbitacins in cucumber (*Cucumis sativus*), melon (*Cucumis melo*), and watermelon (*Citrullus lanatus*) [17, 18]. Cucurbitacins are triterpenoids that confer a bitter taste in Cucurbitaceae plants [17]. The distinct cucurbitacins in cucurbits are structurally similar, and are synthesized mainly by conserved syntenic biosynthetic genes, which were reported to be regulated by a cluster (hereafter referred to as the *Bt* cluster) harboring several *bHLHs* [17, 18]. In cucumber, *Bt* (*Bitter fruit*) and *Bl* (*Bitter leaf*), located in the *Bt* cluster, can regulate the accumulation of cucurbitacin C (CuC) in fruits and leaves, respectively [17]. In melon and watermelon, the syntenic homologs of the *Bt* cluster were found to exert similar functions in diverse tissues by regulating cucurbitacin B (CuB) in melon and cucurbitacin E (CuE) in watermelon [18]. However, the evolutionary history and functional divergence of genes within the *Bt* cluster are largely unknown.

In this study, we identified and characterized 1,160 *bHLH* genes and performed a comparative evolutionary analysis in the seven cucurbit crops: cucumber, melon, watermelon, bottle gourd (*Lagenaria siceraria*), wax gourd (*Benincasa hispida*), bitter gourd (*Momordica charantia*), and pumpkin (*Cucurbita pepo*). We determined orthologous and paralogous relationships among these *bHLH* genes and investigated features of *bHLH* tandem-duplicated genes (TDGs). Furthermore, we described the evolutionary history and divergence of the *Bt* cluster and reported the discovery of a novel functional *bHLH* gene regulating cucurbitacin biosynthesis in a *Bt* paralogous cluster. These results enhance our understanding of the biosynthetic regulation of secondary metabolites through TFs.

## Results

### Identification and classification of *bHLH* genes in seven cucurbit crops

By searching the genomes of seven cucurbits we identified 149 non-redundant *bHLH* genes in cucumber, 151 in melon, 154 in watermelon, 155 in bottle gourd, 150 in wax gourd, 150 in bitter gourd, and 251 in pumpkin. All *bHLH* genes were numbered according to their genomic coordinates, yielding *CsabHLH001–149*, *CmebHLH001–151*, *ClabHLH001–154*, *LsibHLH001–155*, *BhibHLH001–150*, *MchbHLH001–150*, and *CmabHLH001–251* (Supplementary Data Table S1). The number of *bHLH* genes was comparable among the cucurbit species investigated here, with the exception of pumpkin, whose genome encoded 1.67-fold more bHLH family members than the average number observed in the other six cucurbit crops, possibly due to a lineage-specific recent WGD event [8].

**Figure 1:**
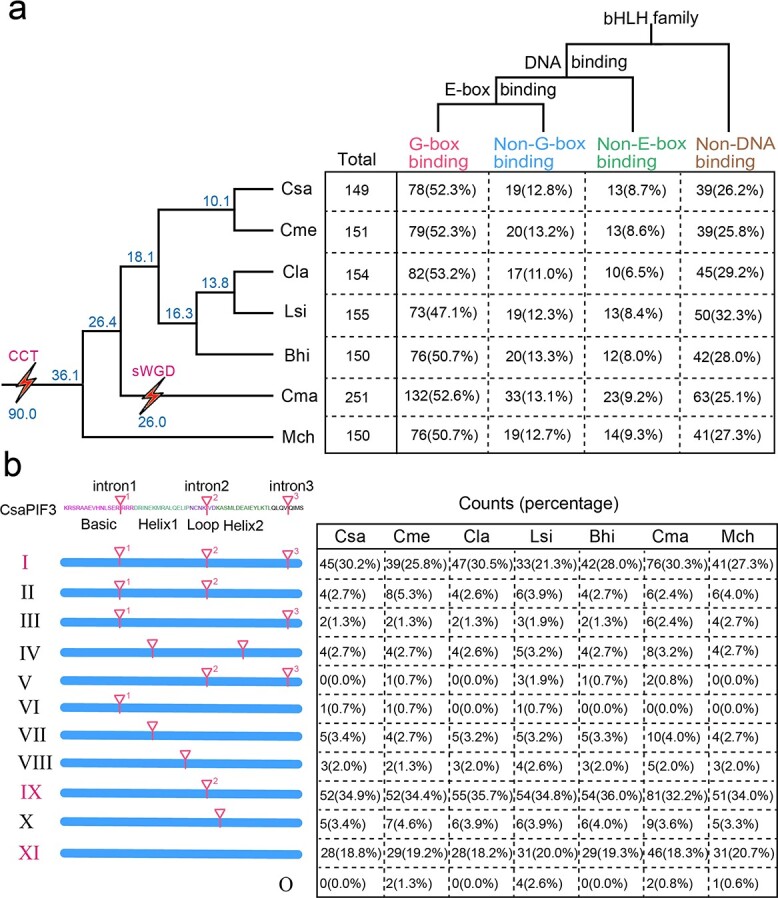
DNA-binding ability and conserved intron distribution patterns of *bHLH* genes in seven cucurbit crops. **a** Predicted DNA-binding characteristics based on the amino acid sequences of the bHLH domain. A species tree of seven cucurbits is shown on the left. Csa, cucumber; Cme, melon; Cla, watermelon; Lsi, bottle gourd; Bhi, wax gourd; Cma, pumpkin; Mch, bitter gourd; sWGD: specific whole-genome duplication. Blue numbers show the estimated divergence time of each node or occurrence time of WGD. **b** Intron distribution patterns within the bHLH domain of seven cucurbit crops. The positions of introns are indicated by triangles and numbered 1–3 based on the bHLH region of *CsaPIF3* (*CsaV3_2G007370*), which is shown at the top. The count and percentage of bHLHs displaying each pattern in seven cucurbit crops are given in the table on the right.

The basic region in the bHLH domain determines its DNA-binding activity [20]. Using previously published criteria [5], the bHLH TFs were divided into one group of DNA-binding proteins and another group of non-DNA-binding proteins (25.1–32.3% of all bHLH TFs). Furthermore, we subdivided the DNA-binding bHLH proteins into two subcategories as a function of their predicted cognate DNA-binding motif: E-box- and non-E-box-binding proteins (8.0–9.3% of all bHLH TFs). E-box-binding bHLH proteins were further subdivided into two groups: G-box-binding proteins (47.1–53.2% of all bHLH TFs) and non-G-box-binding proteins (11.0–13.3% of all bHLH TFs) (Fig. 1a).

Intron and exon structures can help elucidate the phylogenetic relationships of a gene family. We identified 12 different intron distribution patterns based on the number (zero to three) and relative positions of introns within the region encoding the bHLH domain (designated I–XI and O; Fig. 1b). About 80% of the identified bHLHs featured the three most common patterns: I, IX, and XI. Pattern IX was the most common, with only one intron in the loop region. Pattern I was characterized by three introns at three highly conserved positions and was the second most common pattern. Finally, pattern XI, with no intron in the bHLH domain-encoding region, was the third most common pattern (Fig. 1b). The intron distribution patterns in cucurbit crops are like those in tomato (*Solanum lycopersicum*) [21] and arabidopsis (*Arabidopsis thaliana*) [5], suggesting that this might be a common feature among plant species.

### Evolution of *bHLH* genes in seven cucurbit crops

**Figure 2:**
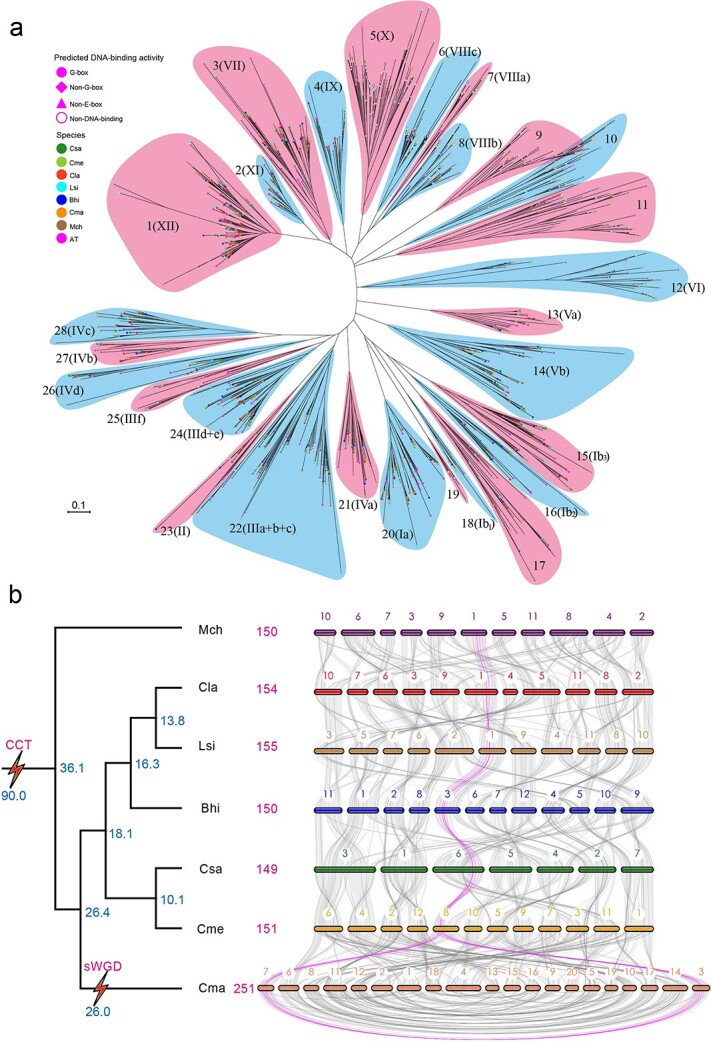
Evolution of *bHLH* genes in seven cucurbit crops. **a** Unrooted neighbor-joining phylogenetic tree of 1,322 *bHLHs* from cucumber, melon, watermelon, bottle gourd, wax gourd, pumpkin, bitter gourd, and arabidopsis. *bHLHs* are divided into 28 subfamilies. The Roman numerals in brackets denote subfamilies, as defined in arabidopsis. Detailed information on the phylogenetic tree is given in Supplementary Data Fig. S1. **b** Synteny analysis of *bHLH* genes among seven cucurbit crops. A species tree of seven cucurbits is shown on the left. sWGD, specific whole-genome duplication. Blue numbers are estimated divergence times (in millions of years ago) of each node or occurrence time of WGD. Magenta numerals are the numbers of *bHLH* genes in each cucurbit crop. Magenta and gray lines display the collinear *bHLH* genes among seven cucurbit crop genomes. The light gray lines denote collinear blocks.

To understand the phylogenetic relationships among *bHLHs* in cucurbit crops, we constructed an unrooted neighbor-joining phylogenetic tree using a multiple alignment of the bHLH domain. The 1,160 cucurbit *bHLH* genes are clustered into 28 subfamilies according to the classification of arabidopsis *bHLHs* [22] (Fig. 2a;Supplementary Data Fig. S1). In most subfamilies, DNA-binding properties, intron pattern distributions, and motif architectures of *bHLH* genes were relatively conserved (Supplementary Data Fig. S1), suggesting the reliability of the classification and phylogenetic tree of cucurbit *bHLH* genes presented here.

To further investigate the evolutionary trajectory of the bHLH family, we constructed a syntenic map and generated a syntenic gene list of *bHLH* genes across the seven cucurbit crops (Fig. 2b; Supplementary Data Table S2). Most highly conserved syntenic blocks were shared by the seven cucurbit genomes, with ~90% of all *bHLH* genes mapping to these orthologous blocks. For instance, >10 orthologous *bHLH* genes formed a superblock that was perfectly matched in all seven cucurbit crop genomes, as represented by the magenta lines in Fig. 2b. We next clustered and identified 151 *bHLH* orthologous groups (OGs) present in at least two cucurbit genomes (Supplementary Data Table S2, OG001–151) and 13 species-specific *bHLH* OGs (Supplementary Data Table S2, OG152–164). In 92% of all OGs, bHLH TFs from each cucurbit crop exhibited the same DNA-binding ability and their encoding genes showed the same intron distribution pattern (Supplementary Data Table S2).

We then identified paralogous gene pairs: 28 pairs in cucumber, 28 pairs in melon, 31 pairs in watermelon, 25 pairs in bottle gourd, 14 pairs in wax gourd, 33 pairs in bitter gourd, and 129 pairs in pumpkin (Supplementary Data Fig. S2 and Supplementary Data Table S3). About 94% of these paralogous bHLH TFs shared the same DNA-binding ability and their encoding genes showed the same intron distribution pattern (Supplementary Data Fig. S2). All paralogous *bHLH* gene pairs had non-synonymous substitutions/synonymous substitutions (*K*_a_/*K*_s_) values <0.6 (Supplementary Data Fig. S3), suggesting that the majority of paralogous *bHLH* genes have undergone purifying selection. These paralogous genes might be the extant product of WGDs during the evolutionary history of cucurbit crops. These results offer novel insights into the evolution of *bHLH* genes in cucurbit crops.

### Characterization of *bHLH* tandem-duplicated genes

Local gene duplication generates TDGs and is ubiquitous during genome evolution [23]. To explore the features and potential functions of *bHLH* TDGs, we identified clusters of *bHLH* TDGs—10 genes in cucumber (four clusters); 9 in melon (three clusters); 14 in watermelon (five clusters); 15 in bottle gourd (seven clusters); 10 in wax gourd (four clusters); 10 in bitter gourd (four clusters); and 8 in pumpkin (four clusters)—based on chromosome localization of the genes and sequence similarity of the encoded proteins (Supplementary Data Fig. S4; Table 1). TDG clusters were named according to their order in their genomic coordinates, such as ‘T1’ and ‘T2’ (Supplementary Data Fig. S4). The numbers of *bHLH* TDGs varied across the seven cucurbit crops, with more *bHLH* TDGs in watermelon and bottle gourd than in the other five species. The *K*_s_ values for each TDG pair belonging to the same cluster were then calculated to trace their divergence time after duplication (Supplementary Data Table S4). In watermelon and bottle gourd, we obtained two (T1 and T5) and three (T4, T5, and T6) gene pairs with *K*_s_ values <0.32, suggesting that these TDGs may emerge recently after the divergence between the two species (Supplementary Data Table S4). This might explain the higher number of *bHLH* TDGs in these two species. Pumpkin, which underwent a recent WGD event, had the smallest number of *bHLH* TDGs, despite having the largest *bHLH* gene family among the seven cucurbits (Table 1; Supplementary Data Fig. S4 and Supplementary Data Table S4). Considering WGD events may accelerate the loss of TDGs [24], we speculate that the evolution of TDGs in pumpkin might have been affected by the recent WGD event [8].

**Table 1:** List of orthologous gene groups of TDGs among seven cucurbit crops

**Orthologous group**	**Melon**	**Cucumber**	**Watermelon**	**Wax gourd**	**Bottle gourd**	**Bitter gourd**	**Pumpkin**
OG001	*CmebHLH104* ^*^ *CmebHLH105* ^*^ *CmebHLH106* ^*^	*CsabHLH125* ^*^ *CsabHLH126* ^*^ *CsabHLH127* ^*^	*ClabHLH009* ^*^ *ClabHLH010* ^*^ *ClabHLH011* ^*^	*BhibHLH041* ^*^ *BhibHLH042* ^*^ *BhibHLH043* ^*^	*LsibHLH007* ^*^ *LsibHLH008* ^*^ *LsibHLH009* ^*^	*MchbHLH004* ^*^ *MchbHLH005* ^*^	*CmabHLH030* ^*^ *CmabHLH031* ^*^ *CmabHLH086* ^*^ *CmabHLH087* ^*^
OG002	*CmebHLH112*	*CsabHLH087* ^*^ *CsabHLH088* ^*^	*ClabHLH049* ^*^ *ClabHLH050* ^*^ *ClabHLH051* ^*^	*BhibHLH048* ^*^ *BhibHLH049* ^*^ *BhibHLH050* ^*^	*LsibHLH013* ^*^ *LsibHLH014* ^*^	*MchbHLH001*	*CmabHLH024 CmabHLH091*
OG003	*CmebHLH120* ^*^ *CmebHLH121* ^*^ *CmebHLH122* ^*^ *CmebHLH123* ^*^	*CsabHLH093* ^*^ *CsabHLH094* ^*^ *CsabHLH095* ^*^	*ClabHLH001* ^*^ *ClabHLH002* ^*^ *ClabHLH003* ^*^ *ClabHLH004* ^*^	*BhibHLH135* ^*^ *BhibHLH136* ^*^	*LsibHLH120* ^*^ *LsibHLH121* ^*^ *LsibHLH122* ^*^ *LsibHLH123* ^*^	*MchbHLH077* ^*^ *MchbHLH078* ^*^	*CmabHLH017 CmabHLH194* ^*^ *CmabHLH195* ^*^
OG004	*CmebHLH082* ^*^ *CmebHLH083* ^*^	*CsabHLH064* ^*^ *CsabHLH065* ^*^	*ClabHLH064* ^*^ *ClabHLH065* ^*^	*BhibHLH008* ^*^ *BhibHLH009* ^*^	*LsibHLH071* ^*^ *LsibHLH072* ^*^	*MchbHLH128*	*CmabHLH231 CmabHLH197*
OG005	*CmebHLH138*	*CsabHLH111*	*ClabHLH079* ^*^ *ClabHLH080* ^*^	*BhibHLH145*	*LsibHLH132*	*MchbHLH146*	*CmabHLH210*
OG006	*CmebHLH037*	*CsabHLH117*	*ClabHLH113*	*BhibHLH058*	*LsibHLH109* ^*^ *LsibHLH110* ^*^	*MchbHLH088*	*CmabHLH054 CmabHLH148*
OG007	*CmebHLH079*	*CsabHLH061*	*ClabHLH061*	*BhibHLH005*	*LsibHLH076* ^*^ *LsibHLH077* ^*^	*MchbHLH052*	*CmabHLH182*
OG008	*CmebHLH039*	*CsabHLH049*	*ClabHLH115*	*BhibHLH057*	*LsibHLH113*	*MchbHLH091* ^*^ *MchbHLH092* ^*^	*CmabHLH057*
OG009	*CmebHLH066*	*CsabHLH036*	*ClabHLH150*	*BhibHLH083*	*LsibHLH148*	*MchbHLH105* ^*^ *MchbHLH106* ^*^ *MchbHLH107* ^*^ *MchbHLH108* ^*^	No gene
OG010	*CmebHLH141*	*CsabHLH016*	*ClabHLH039*	*BhibHLH148*	*LsibHLH025*	*No gene*	*CmabHLH224* ^*^ *CmabHLH225* ^*^
**Number of TDGs (clusters)**	**9 (three)**	**10 (four)**	**14 (five)**	**10 (four)**	**15 (seven)**	**10 (four)**	**8 (four)**

^*^TDGs.

Furthermore, we investigated the distribution of TDGs in OGs and their potential functions. All TDGs were distributed in 10 OGs (Table 1), including four (OG001, OG002, OG003, and OG004) in which we detected TDG clusters in at least four cucurbit crops. Homologous genes in arabidopsis for these TDG cluster genes in OG001 and OG002 were reported to be involved in the regulation of iron homeostasis [25, 26]. OG003 included the *Bt* cluster, which regulates cucurbitacin biosynthesis in different tissues in cucumber, melon, and watermelon [17, 18], whereas the function of genes in OG004 remains to be elucidated in cucurbits.

**Figure 3:**
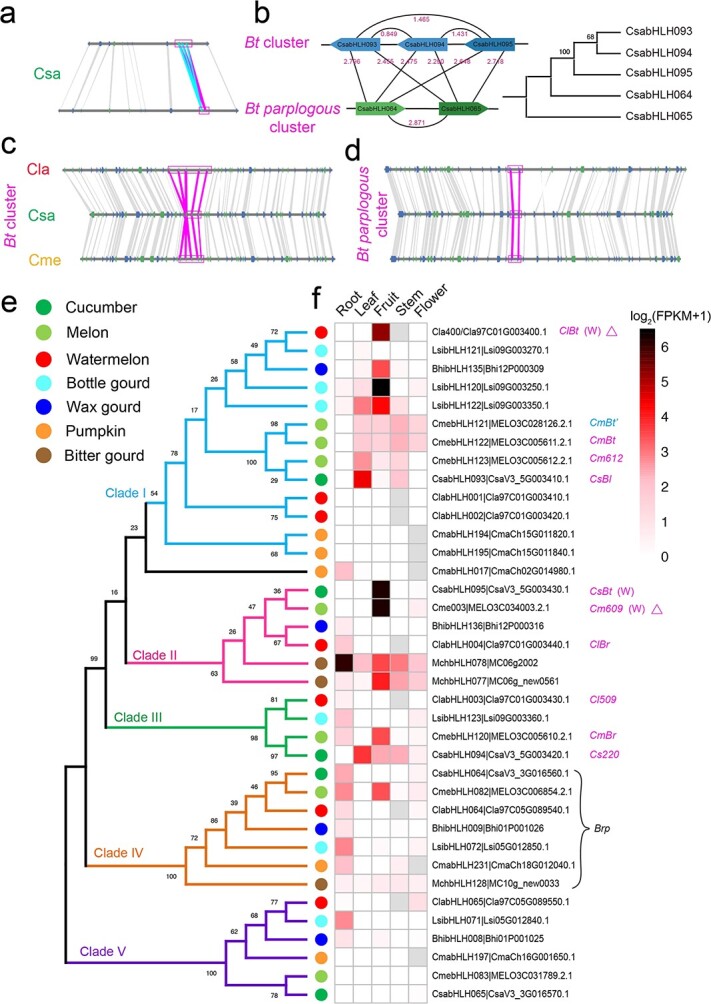
Evolution and divergence of the *Bt* cluster. **a** The syntenic block between the *Bt* cluster and its paralogous cluster in cucumber. **b** Evolution of the *Bt* cluster and its paralogous cluster in cucumber. The magenta numbers are *K*_s_ values. **c**, **d** Syntenic regions of the *Bt* cluster (**c**) and its paralogous cluster (**d**) among cucumber (Csa) melon (Cme), and watermelon (Cla). **e**, **f** Phylogenetic tree (**e**) and expression heat maps (**f**) of genes in the *Bt* cluster and its paralogs. Gray boxes indicate missing values. The magenta ‘W’ means genes only expressed in fruits of wild accessions. The magenta abbreviations are the same as in reference 18. Triangles mean premature translational termination. The CDSs of *CmBt* and *CmBt′* are identical. One gene in the *Bt* paralogous cluster, exhibiting high expression in the roots of cucurbit crops, is designated *Brp*.

### Evolution and divergence of the *Bt* cluster

In cucumber, melon, and watermelon, the *Bt* cluster regulates cucurbitacin biosynthesis in different tissues [17, 18]. To explore the evolution and divergence of this cluster across more cucurbit crops, we performed a comprehensive comparative genomic analysis by assessing local synteny of paralogous or orthologous genes, as well as constructing a phylogenetic tree and analyzing gene expression patterns. Syntenic paralogous gene analysis detected a paralogous cluster (OG004 in Table 1) of the *Bt* cluster in cucurbit crops (Fig. 3a; Supplementary Data Fig. S5 and Supplementary Data Table S5). The *K*_s_ values of each paralogous gene pair mapping to the *Bt* cluster were much smaller than those of all gene pairs between clusters (Fig. 3b; Supplementary Data Fig. S6). The phylogenetic tree showed that the evolutionary distance between genes in the *Bt* cluster is closer than that of genes between clusters (Fig. 3b). These results suggested that the *Bt* cluster in cucurbits might have arisen after the CCT event [7] (Fig. 3b; Supplementary Data Fig. S6). By local syntenic orthologous gene analysis, we found that the *Bt* cluster is collinearly distributed among the Cucurbitaceae crops, as is its paralogous cluster (Fig. 3c and d). In the paralogous cluster, the number of genes appears to be conserved, with two genes in each species (Fig. 3d). However, the gene numbers in the *Bt* cluster were much more flexible, with three genes in cucumber, five genes in melon, five genes in watermelon, four genes in bottle gourd, and two genes in wax gourd in the *Bt* cluster (Fig. 3c; Supplementary Data Fig. S6). To infer the number of ancestral genes in the *Bt* cluster and their evolution, we divided *bHLH* genes from the *Bt* cluster and its paralogous cluster from the seven cucurbit species into five major clades (Clades I–V) based on the phylogenetic tree (Fig. 3e). The genes from the *Bt* cluster were distributed in Clades I, II, and III (Fig. 3e), suggesting three ancestral genes in the *Bt* cluster after the CCT event. In cucumber, three *Bt* cluster genes remained in Clades I, II, and III. However, new members appeared in Clade I for watermelon, melon, and bottle gourd after species diversification, based on the intraspecies and interspecies *K*_s_ values and phylogenetic analysis (Fig. 3e; Supplementary Data Figs S6 and S7). For example, the *K*_s_ values of each pair among *CmebHLH121*, *CmebHLH122*, and *CmebHLH123* in the *CmeBt* cluster were smaller than interspecies *K*_s_ values, suggesting that these three genes arose in melon after its divergence from the other species (Supplementary Data Figs S6 and S7). In addition, several members of Clade II or Clade III were lost in some cucurbit crops: wax gourd lacked a gene in Clade III (Fig. 3e). Finally, we inferred the evolutionary correspondence of *Bt* cluster genes among the seven cucurbit species (Supplementary Data Table S6).

The expression patterns of genes in the *Bt* cluster and its paralogous cluster showed a high degree of tissue specificity. For example, *Cla400*, *BhibHLH135*, *LsibHLH120*, *CsabHLH095* (*CsBt*), *Cme003*, and *MchbHLH077* were predominantly expressed in fruits, while *CsabHLH093* (*CsBl*) exhibited a leaf-specific expression pattern and *CmabHLH017*, *ClabHLH004*, *LsibHLH123*, *CsabHLH064*, and *LsibHLH071* were mainly expressed in roots (Fig. 3f). These expression patterns imply the potential functions of the respective *bHLH* genes in specific tissues, echoing the expression of *CsBt* and *CsBl* in cucumber fruits and leaves and their role in regulating cucurbitacin biosynthesis, respectively [17, 18]. Notably, most genes in Clade IV were highly expressed in roots (Fig. 3f), suggesting their potential roles in regulating cucurbitacin biosynthesis in this tissue.

### A novel functional *bHLH* gene of the *Bt* paralogous cluster regulates cucurbitacin biosynthesis in roots

**Figure 4:**
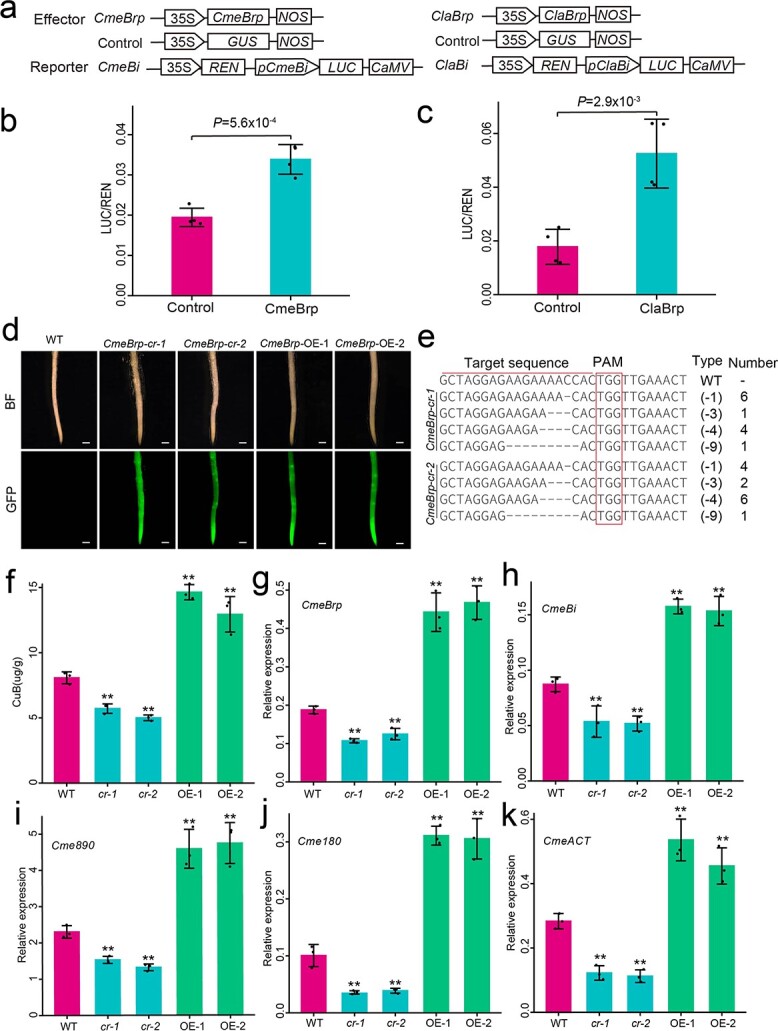
*Brp* participates in regulating cucurbitacin biosynthesis. **a** Schematic diagram showing the constructs used in the transient transcriptional activity assays. **b**, **c***Brp* activates *Bi* expression in a tobacco leaf assay system for melon (**b**) and watermelon (**c**). Values are mean ± standard deviation from four biological replicates. **d** GFP signals detected in hairy roots of *CmeBrp*-overexpressing and *CmeBrp* knockout lines. Scale bar = 1 mm. **e** Sequence analysis of *CmeBrp* in two knockout lines. A total of 25 colonies were sequenced in *CmeBrp-cr* lines. Mutant types and numbers are showed at right. **f** Content of CuB in WT, *CmeBrp* knockout, and *CmeBrp* overexpressing lines. **g** Relative expression of *CmeBrp* in WT, *CmeBrp* knockout, and *CmeBrp*-overexpressing lines. **h***–***k** Relative expression of four key CuB biosynthetic genes, *CmeBi* (**h**), *Cme890* (**i**), *Cme180* (**j**), and *CmeACT* (**k**), in WT, *CmeBrp* knockout, and *CmeBrp*-overexpressing lines. Transcript levels were measured by RT–qPCR. Values are represented as mean ± standard deviation from three biological replicates. **P <.01, Student’s *t*-test.

We found that one gene in the *Bt* paralogous cluster of each cucurbit crop, belonging to Clade IV, exhibited a high level of expression in roots (designated *Brp*; Fig. 3e and f). Given its evolutionary history, we hypothesized that *Brp* might be involved in regulating the biosynthesis of cucurbitacins in the roots of cucurbit crops. To explore the function of *Brp* in cucurbits, we conducted heterologous transient expression experiments in *Nicotiana benthamiana* leaves, using a reporter construct consisting of the promoter from the *Bitter* (*Bi*) locus of melon and watermelon, driving the expression of the firefly luciferase (*LUC*) gene. Here the *Bi* gene encodes oxidosqualene cyclase, which catalyzes the first step of cucurbitacin biosynthesis (Supplementary Data Fig. S8) [18]. Compared with the control, the normalized transcriptional activity [ratio of firefly luciferase to *Renilla* luciferase (LUC/REN)] of pro*Bi*:*LUC* was significantly promoted by transient overexpression of *Brp* from melon (*CmebHLH082*) and watermelon (*ClabHLH064*) (Fig. 4a–c).

To better understand the underlying function of *Brp* in cucurbit crops, we generated *CmeBrp*-overexpressing (*CmeBrp*-OE) transgenic hairy melon roots with *CmeBrp* expression driven by the constitutive cauliflower mosaic virus (CaMV) 35S promoter (Fig. 4d). We also produced transgenic hairy melon roots of CRISPR/Cas9 (*cr*)-mediated *CmeBrp* knockout mutants (*CmeBrp-cr*) (Fig. 4d and e). We then quantified cucurbitacin B (CuB) contents in hairy roots of the wild-type (WT), *CmeBrp-cr*, and *CmeBrp*-OE lines, which revealed a drastic increase in CuB contents in *CmeBrp*-OE lines and a significant decrease in *CmeBrp-cr* lines compared with the WT (Fig. 4f; Supplementary Data Fig. S9). Real-time quantitative polymerase chain reaction (RT-qPCR) analysis indicated that the expression of *CmeBrp* and four key biosynthetic genes of CuB (*CmeBi*, *Cme890*, *Cme180*, and *CmeACT*; Supplementary Data Fig. S8) [18] were markedly upregulated in the *CmeBrp*-OE lines compared with the WT (Fig. 4g–k), whereas the expression of four key biosynthetic genes of CuB were substantially lower in the *CmeBrp-cr* lines relative to the WT (Fig. 4h–k). In summary, these results suggest that *CmeBrp* may regulate the biosynthesis of cucurbitacin B in melon roots.

## Discussion

Comparative genomics analysis can be powerful approaches to provide novel insights into gene function and evolution. In this study, we performed comparative genomics analysis of *bHLH* genes in seven cucurbit species and inferred the evolution and divergence of the *Bt* cluster. Together with evolutionary relationships and expression profiles, these results empowered the discovery of a novel gene regulating cucurbitacin biosynthesis. The methodology applied in this research presents an example of how gene family analysis facilitates functional gene studies.

Tandem duplications are a widespread phenomenon in plant genomes and play significant roles in evolution and adaptation to changing environments [27]. Compared with WGD-derived duplicate genes, TDGs provide a continuous supply of variants available for adaptation to continuously changing environments [28]. Even though the number of *bHLH* genes is the highest in pumpkin compared with that in other cucurbit crops, it has the fewest *bHLH* TDGs. Both TDGs and WGDs can lead to the expansion of gene families and increase gene diversity. During pumpkin evolution, WGD may have provided additional genetic material and increased gene diversity, and thus the fraction of lost tandem arrays was significantly larger than that of lost non-tandem genes [24]. Therefore, we hypothesize that TDGs in pumpkin were dramatically lost after the recent WGD, which might have resulted from a gene dosage effect.

Compared with the metabolic biosynthetic gene clusters [29–32], the TF clusters governing these metabolic enzymes are less identified and characterized [33]. Unlike the metabolic biosynthetic clusters, which include genes encoding various classes of metabolic enzymes [34, 35], TFs in the same cluster may have overlapping or distinct regulatory functions. In Madagascar periwinkle (*Catharanthus roseus*), three clustered *bHLH* genes [named *BIS1* (*bHLH iridoid synthesis 1*), *BIS2*, and *BIS3*] regulate iridoid biosynthesis in the terpenoid indole alkaloid biosynthetic pathway [36–38]. However, the origin, copy number, and evolution of TF clusters are largely unknown [38]. In this study, we determined that gene number within the *Bt* cluster is variable across species, and a novel bHLH cluster consisting of two genes that is relatively conserved and paralogous to the *Bt* cluster also regulates cucurbitacin biosynthesis. These *bHLH* genes belonged to the 15(Ib3) subfamily (Supplementary Data Fig. S1). Although the functions of *AtbHLHs* belonging to the 15(Ib3) subfamily were unknown in arabidopsis, these *AtbHLHs* clustered with *Brps* into a clade and were highly expressed in roots. Therefore, we speculated that the *Bt* paralogous cluster is an ancestral cluster, as evidenced by the higher *K*_s_ values of each pair of genes in the *Bt* paralogous cluster (Fig. 3b; Supplementary Data Fig. S6). These findings will help in understanding the function and evolution of TF clusters.

## Materials and methods

### Data collection and identification of *bHLH* genes in seven cucurbit crops

Melon genome sequence data were obtained from the Melonomics database (www.melonomics.net), bitter gourd genome sequence data were obtained from https://db.cngb.org/search/assembly/CNA0000004/, and for the other five cucurbit crops (cucumber, watermelon, wax gourd, bottle gourd, and pumpkin) genome sequence data were obtained from http://cucurbitgenomics.org. Information and sequences for *A. thaliana bHLH*s (*AtbHLH*s) were retrieved from https://www.arabidopsis.org. The bHLH proteins of seven cucurbit crops (*CsabHLH*s, *CmebHLH*s, *ClabHLH*s, *BhibHLH*s, *LsibHLH*s, *CmabHLH*s, and *MchbHLH*s) were predicted using the HLH hidden Markov model (HMM) profile obtained from Pfam (http://pfam.xfam.org, PF00010) and used as queries to search the bHLH proteins from cucurbit crop sequences with HMMER (version 3.1b2) software (http://hmmer.janelia.org). We also performed a BLASTP search against the AtbHLH database. Redundant protein sequences were removed by searching in the NCBI database (https://www.ncbi.nlm.nih.gov/Structure/bwrpsb/bwrpsb.cgi) and the SMART database (http://smart.embl-heidelberg.de). In addition, some *bHLH* genes were re-annotated, due to errors existing in the raw annotation, by *ab initio* prediction, transcript mapping, and evidence from other reference genome versions.

### Multiple alignments and phylogenetic analysis

Multiple sequence alignments of identified bHLH domains of 162 arabidopsis (AtbHLH), 149 cucumber (CsabHLH), 151 melon (CmebHLH), 154 watermelon (ClabHLH), 150 wax gourd (BhibHLH), 155 bottle gourd (LsibHLH), 251 pumpkin (CmabHLH), and 150 bitter gourd (MchbHLH) bHLH proteins were carried out using MUSCLE software with default parameters. To visualize the conserved motifs, the sequences were analyzed with WEBLOGO programs (http://weblogo.berkeley.edu) based on the result of each cucurbit crop multiple sequence alignment. Based on the results of all seven cucurbit crops and arabidopsis multiple sequence alignments, a neighbor-joining tree was constructed using MEGA 7.0 [39] using a bootstrap test with 1,000 replicates based on the Jones–Taylor–Thornton (JTT) model and 80% partial deletion for gap treatment. The phylogenetic tree was visualized in MEGA 7.0.

### DNA-binding ability analysis

Firstly, based on multiple sequence alignments of bHLH domains proteins, we identified conserved amino acid residues in the bHLH domains and four conserved motifs including one basic motif, two amphipathic α-helices, and one loop that linked the two amphipathic α-helices (Supplementary Data Fig. S10 and Supplementary Data Table S7). Then we defined proteins with more than five basic amino acid residues in the basic region as DNA-binding proteins based on the criteria developed by Toledo-Ortiz *et al*. [5]. The bHLH proteins were divided into a group of DNA-binding proteins and a group of non-DNA-binding proteins. Furthermore, the DNA-binding proteins were subdivided into two subcategories, E-box (based on the presence of E-2 and R-4) and non-E-box-binding proteins (without the simultaneous presence of E-2 and R-4). Then E-box-binding proteins were further subdivided into two groups, including G-box-binding proteins (H/K-1, E-2, and R-5 were required) and non-G-box-binding proteins. Consensus amino acids and the numbers of them, such as E-2, are described in Supplementary Data Table S7.

### Gene structure analysis and protein motif detection

The exon/intron organization and splicing phase of the predicted *bHLHs* were investigated based on the GFF/GTF annotation files of seven cucurbit crop genomes. Furthermore, to discover the intron distribution pattern, we did alignment analysis with the coding sequence of the bHLH domain and genome sequences using Blat software [40]. Then exon/intron structures, the splicing phase, and different regions of the bHLH domain were graphically displayed using the Gene Structure Display Server [41] (GSDS, http://gsds.cbi.pku.edu.cn/). To analyze other conserved motifs in the complete amino acid sequences of bHLHs, the protein sequences of candidate bHLHs were analyzed using MEME [42] (version 5.1.1) software (http://meme-suite.org/tools/meme). The parameter settings were: number of motifs to find, 15; minimum width of motifs, 6; maximum width of motifs, 50.

### Identification of orthologous genes among seven cucurbit crops and paralogous genes in each cucurbit crop

Gene synteny analysis by MCscanX [43]) with default parameters. Based on the syntenic blocks, each inter-genomic bHLH orthologous genes among seven cucurbit crops were identified. Then we merged the orthologous gene pair information using a python script. Visualizations of orthologous genes were generated using jcvi (https://github.com/tanghaibao/jcvi). Similarly, intra-genomic *bHLH* paralogous genes were identified. Paralogous genes were graphically displayed as circos figures.

### Chromosomal locations and tandem-duplicated gene detection

The chromosomal positions of bHLH loci were obtained from each cucurbit crop general feature format (GFF) or gene transfer format (GTF) gene structure annotation files. The distribution of *bHLH* genes on chromosomes in each cucurbit crop was drawn using TBtools software [44]. Tandem genes were detected using SynOrths [45] and MCscanX [43] softwares the with default parameters.

### *K*_a_, *K*_s_ and *K*_a_*/K*_s_ analysis

The non-synonymous substitutions(*K*_a_), synonymous substitutions (*K*_s_), and *K*_a_*/K*_s_ values of paralogous gene pairs and tandem array genes (between any two genes in the same tandem-duplicated gene cluster) were calculated using KaKs_Calculator [46].

### Gene expression analyses of *bHLH* genes in seven cucurbit crops

RNA-seq data for the *bHLH* genes were obtained from previous studies of differential gene expression in organs and tissues in seven cucurbit crops [8, 47–52]. All clean RNA-seq reads from each sample were mapped onto the corresponding genome sequences, using Hisat2 [53] (version 2.1.0) with default parameters. The generated BAM format alignments, together with the gene GTF annotation file, were then fed to StringTie (v1.3.4d) software to compute FPKM (the fragments per kilobase of exon model per million reads mapped) values of genes [54] (v1.3.4d). Finally, the log_2_-transformed (FPKM + 1) values were used to generate a heat map by R.

### Dual-luciferase assay

The full-length coding sequence (CDS) of *Brp* was inserted into the pBI121 plasmid to generate the *Brp*-pBI121 effector, while the 2000-bp sequence upstream of the translation initiation start site of the *Bi* gene was cloned into the pGreen II 0800-LUC to generate p*Bi*-LUC double-reporter vector. The *Brp*-pBI121 effector and p*Bi*-LUC reporter vector were transformed into *Agrobacterium tumefaciens* strain GV3101 and GV3101(pSoup-p19), respectively. The reporter and effector were co-infiltrated into *N. benthamiana* leaves at a volume ratio of 9:1. The empty pBI121 vector was used as control. The leaf samples were collected within 60 hours after injection to measure luciferase activities using the Dual-Luciferase Reporter Assay System (Promega, USA, E1910) using a GloMax 20/20 Luminometer (Promega, USA) according to the manufacturer’s instructions (Promega, USA). The relative reporter gene expression levels were expressed as the LUC/REN ratio. Four independent transformations for each sample were performed.

### *Agrobacterium rhizogenes*-mediated hairy root transgenic system in melon

To construct the *CmeBrp* overexpression vector, the full-length CDS of *CmeBrp* was cloned into the pCAMBIA1305.4 binary vector, using an In-Fusion HD Cloning Kit (Clontech). To generate the CRISPR/Cas9 editing vector, the 19-bp sgRNA fragment of *CmeBrp* was assembled into the vector pBSE402 by using the Golden Gate cloning method. The individual read frame green fluorescent protein (GFP) was used as a reporter gene by the constitutive CaMV 35S promoter in *CmeBrp*-OE and *CmeBrp-cr* vectors. These vectors were transformed into *A. rhizogenes* Ar.Qual (catalog number AC1060).

Peeled melon seeds were sterilized with 75% (v/v) ethanol for 30 seconds, followed by 0.3% (v/v) sodium hypochlorite solution for 15 minutes. The sterilized seeds were germinated on MS30 medium at 28°C for 2 days in darkness before the seedlings were grown at 25°C for 1 week under a 16-h light/8-h dark photoperiod until the cotyledons were fully expanded. Cotyledons were cut off at the basal and tip ends, and soaked in diluted *A. rhizogenes* strain containing the binary vector at 28°C for 20 minutes, then co-cultured on MS solid medium for 2 days in darkness at 23°C. To regenerate roots, explants were transferred to MS solid medium containing 100 mg/l Timentin for 2 weeks at 25°C under a 16-h light/8-h dark photoperiod. Positive roots with GFP fluorescence at ~2 cm length were collected for subsequent experiments. In order to meet the dosage of samples and consistency of biological replications in subsequent experiments, we had to mix four or five roots as a single biological replication.

### Ultraperformance liquid chromatography analysis of cucurbitacins B from melon

Ultraperformance liquid chromatography (UPLC) analysis of CuB was performed as described by Zhou *et al*. [18]. Samples were flash-frozen in liquid nitrogen and ground to powder. The resulting powder (0.1 g) was added to methanol (1 ml) and homogenized for 15 min, followed by centrifugation at 10 000 g at 4°C for 10 minutes. The solution was filtered through a 0.22-μm membrane prior to injection and then analyzed on an HPLC system.

### Real-time quantitative PCR

Total RNA was isolated from hairy roots of WT, *CmeBrp*-*cr*, and *CmeBrp*-OE lines using a Quick RNA Isolation Kit (Huayueyang), and samples of 1 μg were reverse-transcribed using a GoScript™ Reverse Transcription Mix, Oligo(dT) (Promega, A2791, USA) according to the instruction manual. Then a quantitative PCR assay was performed on an ABI 7900 (Applied Biosystems) machine using GoTaq^®^ qPCR Master Mix Kit (Promega, A6001, USA) according to the manufacturer’s instructions. Three independent biological replicates were performed. Relative gene expression was determined using the 2^−△CT^ method and the *ubiquitin* gene (*MELO3C009513*) was used as the reference gene [54]. Primers are listed in Supplementary Data Table S8.

## Acknowledgements

This work was supported by the National Natural Science Foundation of China (32130093 to Z.Z., 31772304 to Z.Z.) and the National Key R&D Program of China (2016YFD0100307). This work was also supported by the Taishan Scholar Foundation of the People’s Government of Shandong Province and the Chinese Academy of Agricultural Science (ASTIP-CAAS and CAAS-XTCX2016001), the Shenzhen Municipal (The Peacock Plan KQTD2016113010482651), and the Central Public-interest Scientific Institution Basal Research Fund (No. Y2017PT52).

## Author contributions

Y.X. participated in the design of the research, performed the data analysis, and wrote the manuscript. H.Z. designed the experiments. Y.X., Y.Z. and N.J. performed the experiments. X.Z. and Q.Z. collected genome and RNA sequencing data. H.L. and S.C. revised the manuscript. Z.Z. conceived and designed the research, and revised the manuscript. All authors read and approved the manuscript.

## Data availability

All data supporting the results of this article are included within the article and its additional files.

## Conflict of interests

The authors declare that they have no conflict of interest.

## Supplementary data

Supplementary data is available at *Horticulture Research* online.

## Supplementary Material

Web_Material_uhac038
