# Supplementary material for: Comparative genomics analysis of bHLH genes in cucurbits identifies a novel gene regulating cucurbitacin biosynthesis
Source: Hortic Res. 2022 Feb 19;9:uhac038. doi: 10.1093/hr/uhac038 (PMC9071377; doi:10.1093/hr/uhac038)
Supplement: Web_Material_uhac038 [file web_material_uhac038.zip › Supplemental figures.docx]

**Figure S1. Phylogenetic relationships and characters information of *bHLHs* in seven cucurbits and Arabidopsis. a.** Neighbor-joining phylogenetic tree of *bHLHs*. Different shapes mean different DNA-binding types protein and different colors mean different species; **b.** Expression profiles of *bHLHs* in five tissues. Fruit means silique in Arabidopsis and grey box means missing value; **c.** Gene structure features and intron distribution patterns of *bHLHs*. For better visualization and comparison, all introns were displayed in the same length; **d.** Predicted conserved protein motifs of *bHLHs*. Conserved protein motifs of all *bHLHs* were predicted using meme software in two parts due to the maximum number was 1000 sequences for meme software. The first part was protein sequences in subfamilies 1-14 and the second part was ones in subfamilies 15-28.

Figure continued

**Figure S2. Paralogous gene pairs in cucumber, melon, watermelon, bottle gourd, wax gourd, bitter gourd and pumpkin.** Different color names mean different DNA-binding activity bHLH TFs: G-box-binding (magenta), non-G-box-binding (blue), non-E-box-binding (green) and non-DNA-binding proteins (camel). if the paralogous gene pair was same DNA-binding type, they linked with corresponding color line, otherwise linked with black line. Roman numerals correspond to the intron patterns shown in Fig. 1b. Csa: cucumber, Cme: melon, Cla: watermelon, Lsi: bottle gourd, Bhi: wax gourd, Cma: pumpkin and Mch: bitter gourd.

**Figure S3. The *Ka*/*Ks* values of paralogous gene pairs.** Csa: cucumber, Cme: melon, Cla: watermelon, Lsi: bottle gourd, Bhi: wax gourd, Cma: pumpkin and Mch: bitter gourd.

Figure continued

Figure continued

Figure continued

**Figure S4. Chromosomal localizations and tandem duplication of *bHLH* genes.** Different color names mean different DNA-binding activity bHLH TFs: G-box-binding (magenta), non-G-box-binding (blue), non-E-box-binding (green) and non-DNA-binding proteins (camel). Roman numerals correspond to the intron patterns shown in Fig. 1b. Tandem duplication genes are represented by boxes with purple outlines. TDG clusters are named according to their order in their genomic coordinates, such as “T1”, “T2”. Csa: cucumber, Cme: melon, Cla: watermelon, Lsi: bottle gourd, Bhi: wax gourd, Cma: pumpkin and Mch: bitter gourd.


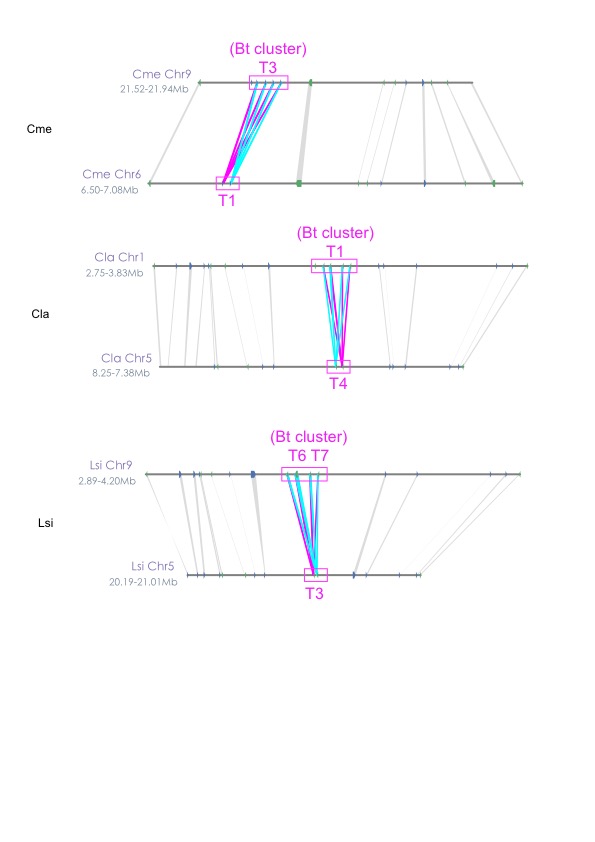


**Figure S5.** **Syntenic block between *Bt* cluster and its paralogous cluster.** The “T1”, “T3”, “T4”, “T6” and “T7” mean TDG cluster names as shown in Figure S5.

**Figure S6. *Ks* values between(among) *Bt* cluster and its paralogous cluster genes.** The magenta numbers were *Ks* values. The dotted line implied that the two genes were not adjacent in bottle gourd and wax gourd.

**Figure S7. *Ks* values of interspecies for *Bt* cluster genes.** The magenta numbers were *Ks* values. The dotted line implied that the two genes were not adjacent in bottle gourd and wax gourd.

**Figure S8. Biosynthetic pathway of cucurbitacin B and its regulating genes in melon. a.** Biosynthetic pathway of cucurbitacin B (CuB). The first step of CuB biosynthesis is that the cyclization of 2, 3-oxi-dosqualene is catalyzed by a cucurbitadienol synthase (CmeBi encoding) to generate cucurbitadienol. Next, cucurbitadienol is catalyzed by one cytochrome P450 enzymes (Cme890) to sequentially generate 11-carbonylcucurbitadienol and 11-carbonyl-20β-hydroxycucurbitadienol. Then 11-carbonyl-20β hydroxycucurbitadienol is oxidized by Cme180 to produce 11-carbonyl-2β,20β-dihydroxycucurbitadienol. Cme180 oxidation is a unique biochemical step in melons, which is different to the synthesis pathway of Cu C in cucumber. the final step comprises the acetylation of CuD by CmACT to generate CuB. **b.** *CmeBt* cluster and CuB biosynthetic genes cluster. There are five genes (*Cme609*, *CmeBr*, *CmeBt*, *CmeBt’*, and *Cme612*) in *CmeBt* cluster. *CmeBr*, *CmeBt*, *CmeBt’*, and *Cme61* are bHLH transcription factors, *Cme609* is premature translational termination in DHL92 melon reference genome. The CDs sequences of CmBt and CmBt' are identical. Six CuB biosynthetic genes comprising *Cme160*, *Cme170*, *Cme180*, *CmeBi*, *CmeACT*, *Cme710* form a metabolic gene cluster in chromosome 11. (modified from ref. 18)

**Figure S9. The ultra performance liquid chromatography (UPLC) analysis of CuB in hairy roots of WT, *CmeBrp* knockout and *CmeBrp* over-expression lines.**

**Figure S10. Conserved amino acid analysis of bHLH domains.** 22 amino acid residues were highly conserved (>50% consensus ratio at least four cucurbit crops).
