# Supplementary material for: Comparative genomics analysis of bHLH genes in cucurbits identifies a novel gene regulating cucurbitacin biosynthesis
Source: Hortic Res. 2022 Feb 19;9:uhac038. doi: 10.1093/hr/uhac038 (PMC9071377; doi:10.1093/hr/uhac038)
Supplement: Web_Material_uhac038 [file FigureS1_Phylogenetic relationships and characters information of bHLHs in seven cucurbits and Arabidopsis.pdf › FigureS1_Phylogenetic relationships and characters information of bHLHs in seven cucurbits and Arabidopsis.pdf]

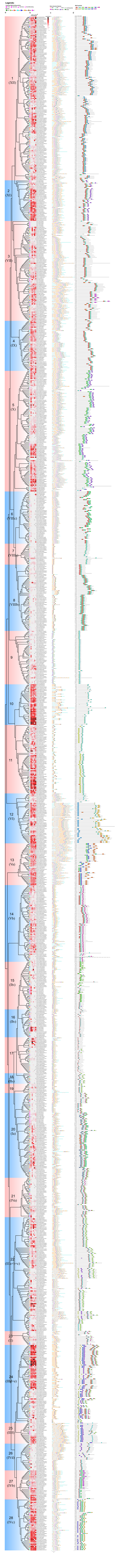
Legends:
Predicted DNA-binding activity
G-box
Non-G-box
Non-E-box
Non-DNA-binding
Species
Csa
Cme
Cla
Lsi
Bhi
Cma
Mch
AT
Gene structure features
CDS
UTR
Intron
0 1 2: splicing phase
Basic
Helix1
Loop
Helix2
Motif symbols
a
b
c
d
root
leaf
fruit
stem
flower
log2(FPKM+1)
p-value
Motif Locations
1
(XII)
